# Laboratory Aspects Relating to the Detection and Prevention of Frailty

**Published:** 2010

**Authors:** Hans-Jürgen Heppner, Jürgen M. Bauer, Cornel C. Sieber, Thomas Bertsch

**Affiliations:** 1 Department of Internal Medicine 2, Nuremberg Clinic, Chair of Internal Medicine V, Geriatrics and Institute for Biomedicine of Ageing, Friedrich Alexander University Erlangen- Nuremberg, Nuremberg, Germany; 2 Institute of Clinical Chemistry, Laboratory Medicine and Transfusion Medicine, Nuremberg Clinic, Germany; * Contributed equally

**Keywords:** Frailty, Laboratory diagnostics, Vitamins, Hormones, Cytokines

## Abstract

Frailty, as a geriatric syndrome, is characterised by the increased vulnerability of the elderly person to internal and especially external stressors. The aim of laboratory diagnostics in the context of the concept of frailty is to record the conditions which encourage the development of frailty, in order to improve these conditions through individual measures or to avoid them for the purpose of preventing frailty. After a presentation of the features of laboratory diagnostics in old age, this article examines haematological aspects, the importance of an adequate vitamin supply, particularly of vitamin D and the adequate description of endocrine functions.

## FEATURES OF LABORATORY DIAGNOSTICS IN OLD AGE

**Figure 1:**
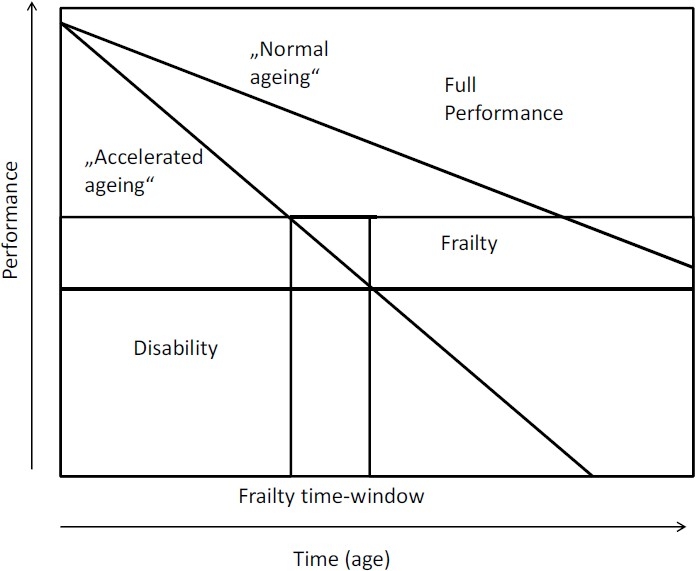
Trajectories of performance over the lifespan. The two curves represent “normal” ageing and “accelerated ageing” (modified after 4).

Frailty is a geriatric syndrome which is characterised by increased vulnerability of the elderly person to internal and especially external stressors (including mitochondrial oxidative stress, diseases, side effects of drugs, mental and social stresses). It is closely associated in its development with age-related loss of muscle mass, sarcopenia, and is accompanied by an increased risk of loss of independence, hospitalisation and mortality.^1^,^2^ The various stages of frailty form a continuum between complete independence on one side and the presence of a disability on the other (Figure 1). Early stages of frailty can only be detected with the aid of specific tests, while later stages are clinically apparent as a result of significant losses of function. The Fried definition for the phenotypic character of frailty proposes five items: weight loss, exhaustion, weakness, slow walking speed and low levels of physical activity (Table 1). Frailty is diagnosed when at least three criteria are met. An individual is said to be prefrail when one or two of these criteria are present. Based on the results of several recent studies, the weight loss criterion may be corrected to weight change as higher BMI values above 30 kg/m^2^ were associated with a loss of functionality which may be an expression of becoming frail. For the calculation of the Frailty Index by Rockwood, it is necessary to count the deficits that are present in an individual.^3^ In the most extensive study, Rockwood and co-workers have published yet 70 deficits that were used for evaluation. These included active diseases, disabilities in the activities of daily living and physical signs from the clinical and neurological examinations. The clinical applicability of these two measures has to be regarded differently. While the Fried criteria concentrate on the physical aspects of frailty and are more easy and quick to work with, the Frailty index by Rockwood incorporates a more diverse spectrum of information on the elderly individual and therefore requires, a more elaborate work up. Although there are scores for the description of the frailty syndrome, Ferrucci and co-workers stated^4^: “The definition of frailty is at an early stage. Recent studies suggest that the frailty syndrome is a physiological state of susceptibility that places older individuals at high risk of rapid deterioration of health and functional status. Multiple medical conditions, age and disease may contribute to the activation of a cyclic, self-sustained pathophysiological pathway that, over the short term, causes physical impairment, functional limitation and severe disability. The factors that contribute to this cyclic metabolic pathway, currently defined as the frailty syndrome, are still unclear and, therefore, there is still uncertainty on what circulating molecules should be considered as biomarkers of frailty”. Although there are a lot of candidates for biomarkers, we focus our review article on the description of laboratory parameters which are available in most clinical routine laboratories. So that with the means of these easily available laboratory parameters, an alteration of function in the organ systems can be detected and the occurrence of the frailty syndrome might be decelerated. Only at the end, we give an outlook to new biomarkers from the cytokine family.

**Figure 2:**
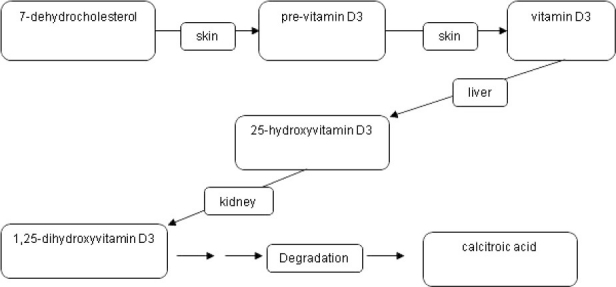
Main creation and degradation pathways in the vitamin D system.

**Table 1:** Criteria for the phenotypic definition of frailty developed by Fried et al.^1^

Weight loss	>5 kg/year
Exhaustion	Depression scale CES-D (2 points)
Weakness	Grip strength (lowest 20%)
Gait speed	5 m (slowest 20%)
Low physical activity	Kcal/week (lowest 20%)
Diagnosis of frailty	3 or more criteria met
Diagnosis of prefrailty	1-2 criteria met

The aim of laboratory diagnostics in everyday medical practice in relation to frailty syndrome is to record predisposing factors for the development of the frailty syndrome, in order to make it possible to introduce the appropriate therapy. This should prevent the functional slide into an advanced stage of frailty and the development of a manifest disability. Essentially, there are two approaches in laboratory medicine: on the one hand, the patients’ results are compared at a certain point in time with the so-called “normal values” or “reference ranges” (cross-sectional assessment, screening). On the other hand, it is also possible to assess the changes in a value measured in the laboratory over the course of time in one patient. This is then referred to monitoring. Establishing reference intervals in Geriatrics is essentially difficult, since in the course of life a variety of noxious substances specific to the individual (alcohol, tobacco, inflammations, toxins etc.) result in a quite individual change in the body’s bio-chemistry and its physiological properties.^5^,^6^ Nevertheless, an attempt was made to establish reference intervals as a guide for the most varying values measured in the laboratory, which are helpful as a working basis. The book *Geriatric Clinical Chemistry* by Faulkner and Meites^7^ is recommended, which lists method-oriented established values measured in the laboratory graded according to the age in years. However, the limitation mentioned above should always be borne in mind when interpreting the laboratory values of the elderly. Because of the age-associated changes and the associated difficulties in comparing them with reference intervals, laboratory diagnostics of the elders should concentrate not so much on identifying a sick person from a collective of essentially healthy people, but rather on evaluating the dynamics of the pattern of laboratory values. This dynamic approach would also make it possible to give consideration to the kinetics and the consequences of the “ageing process”. The dynamic approach means also an “individualistic approach” is needed where individual factors and stressors that occurred over lifetime should be taken into account to develop specific prevention strategies which are adapted to the patient and its present situation. There now follows a discussion of a few special measured values which play a specific role in the context of frailty syndrome.

## DIAGNOSIS OF ANAEMIA

According to the definition of the World Health Organisation, anaemia is present when the haemoglobin concentration in blood in women is less than 12 g/dL and in men less than 13 g/dL. Based on these criteria, the prevalence of anaemia in over 65-year-olds in various populations is between 8 and 44%.^8^–^12^ In a major US study, the prevalence in over 80-year-olds was 26% in men and 20% in women.^13^ In the vast majority of all cases, there are mild degrees of anaemia. The presence of anaemia is associated in the elderly with a marked increase in mortality. This has been demonstrated both for elderly people living independently and for the population of retirement homes.^14^,^15^ In the presence of certain comorbidities such as chronic heart failure or cancer, anaemia causes an added increase in mortality.^16^ Anaemia in the elderly is associated with an increased likelihood of deterioration in their physical capability and loss of their independence.^17^,^18^ Furthermore, a link has been established with the frequency of falls.^19^ Chaves and colleagues also demonstrated a direct link in elderly women between the evidence of anaemia and the presence of frailty.^20^ This link was apparent even in low normal Hb values. Although all these studies demonstrated an association between anaemia and frailty, a causal relationship between these two entities has not yet been adequately established. In many cases of simultaneous frailty and anaemia, it is highly likely that the latter is an epiphenomenon. Nevertheless, clarifying the cause of anaemia in old age is of important clinical significance.

The causes of anaemia in old age are varied. Special mention should be made of anaemia in chronic diseases such as malignancies, chronic infections, collagenosis, chronic inflammation, heart failure and diabetes mellitus. This form of anaemia is the most common form in old age. Around one third of all cases of anaemia in old age are caused by nutritional factors. Here, iron deficiency is of particular significance.^12^ The most important criterion to distinguish anaemia caused by chronic inflammation and that caused by iron deficiency is the serum concentration of the iron storage protein, ferritin, which is lowered as a sign of lack of storage iron in iron deficiency anaemia, and raised in anaemia of chronic diseases. One reason for the increase in ferritin is its capacity to react in inflammatory processes as a so-called acute-phase protein, similarly to the C-reactive protein. This form of anaemia is characterised by well-filled macrophagocytic iron stores, which cannot however be used in sufficient measure for haemoglobin synthesis.^21^ Another cause of anaemia which is avoidable with adequate vitamin substitution is vitamin B12 or folic acid deficiency anaemia. As a result of impaired DNA metabolism, macrocytic, hyperchromic anaemia develops, which is characterised by an increased mean erythrocyte volume with a simultaneously high haemoglobin content of the erythrocytes. Matteini and coworkers found in a cross-sectional study of baseline measures from the combined Women’s Health and Aging Studies that vitamin B12 deficiency may contribute to the frailty syndrome in community-dwelling older women. For prevention of the frailty syndrome, adequate vitamin B12 substitution seems to be important.^22^

Although the cause of anaemia in old age cannot always be reliably explained in every case, nevertheless a few simple laboratory tests allow the causes to be narrowed down as far as possible by differential diagnosis (Table 2). The practical impact of the aforementioned “kinetic approach” can be explained by a study of Ferruci et al.^23^ This study demonstrated that patients with low testosterone levels had a higher risk to develop anaemia within 3 years. As a consequence, in patients with low testosterone serum levels, the haemoglobin values should be monitored.

**Table 2:** Causes of anaemia in old age with the relevant values measured in the laboratory to narrow down the causes (main findings).

Anaemia	Value measured in the laboratory
Chronic inflammatory diseases	Ferritin 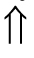
Dietary iron deficiency	Ferritin 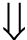
Vitamin B12 and folic acid deficiency	MCV 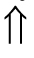 , vitamin B12 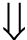 , folic acid 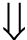
Chronic lymphatic leukaemia	Blood count
Myelodysplastic syndrome	Examination of bone marrow

## VITAMIN DETERMINATION

The importance of vitamin B12 and folic acid in the development of anaemia has already been explained in the previous section. Another vitamin which is attracting increasing attention in terms of its importance for the functionality of the elderly is vitamin D3. Previtamin D3 is produced by the UV radiation from sunlight by photolytic conversion of 7-dehydrocholesterol; this previtamin is converted into vitamin D3 (cholecalciferol) by thermal isomerisation.^24^ The 25-hydroxyvitamin D3 is then formed in the liver by the cytochrome-system by means of 25-hydroxylation.^25^ This step is weakly regulated and correlates with the vitamin D3 intake or synthesis. For this reason and because of its high concentration, 25-hydroxyvitamin D3 compared with the active 1,25-dihydroxyvitamin D3 is a good indicator of the vitamin D3 status.^26^ The actually biologically active 1,25-dihydroxyvitamin D3 is produced by the 1-alpha- hydroxylation in the kidney (Figure 2). The transport protein megalin is responsible for the renal uptake of 25-hydroxyvitamin D3.^27^ The lipophilic 1,25-dihydroxyvitamin D3, bound to a transport protein, reaches the target organs from the kidney. This 1-alpha-hydroxylation step takes place not only in the kidney, also in many other body cells (mammary gland cells, prostate cells, colon cells, ß-cells of the pancreas, cells of the immune system) (Table 3).^28^ The 1,25-dihydroxyvitamin D3 produced in these cells works mainly locally using autocrine and paracrine mechanisms to inhibit cell proliferation, facilitate cell differentiation and immunoregulation (Table 3). The hormone is degraded by 24- hydroxylation, which results in side-chain shortening. The 1,25-dihydroxyvitamin D3 develops its action, like the classic steroid hormones, via an intracellular receptor which, filled with 1,25- dihydroxyvitamin D3, then binds to the DNA and there modifies the gene transcription rate, after interaction with various co-factors.^29^ However, non-genomic rapid effects, as with steroid hormones, can also be demonstrated.^30^ The most important task of the vitamin D system is the regulation of calcium metabolism. The 1,25-dihydroxy vitamin D3 in the intestinal cells brings about the induction of transport proteins, which enable calcium to be absorbed in the intestine.^31^ As a result of the synergistic action of 1,25-dihydroxyvitamin D3 on the osteoblasts and osteoclasts, this enterally absorbed calcium is incorporated into the skeleton and thus bone mineralization is stimulated. However, vitamin D also works at other switch points of calcium homeostasis, such as in the kidney. The close connection between the vitamin D system and calcium or bone metabolism also explains the causal connection between vitamin D deficiency and rickets in children or osteomalacia in adults. In recent years, there have been increasing indications that the vitamin D system plays an important role in the metabolism of a wide variety of organ cells in a number of functional levels such as apoptosis; and that vitamin D3 deficiency is associated with diseases such as hypertension, diabetes mellitus, and various cancers (prostate cancer, breast cancer, colon cancer). Recommended reviews on this topic have been written by Dusso and colleagues,^32^ Grant and Holick^33^ and also Adams and Hewiston.^34^ The significance of vitamin D for these diseases becomes even more important in view of the fact that a large proportion of the population in Europe and North America has a marked 25-hydroxyvitamin D3 deficiency.^35^,^36^ In addition to the general habit of “staying out of the sun” for melanoma prophylaxis and a working life increasingly spent indoors, dietary factors and increasing air pollution are held responsible for this deficiency. Another topic under consideration is the provision of vitamin D3 to an increasingly elderly population. In a major study in over 65-year-olds, Trivedi and colleagues^37^ showed that the additional intake of 300,000 IU vitamin D3/year reduced the fracture rate in the lower arm, spine and femur neck by more than 33%. The fact that vitamin D3 is not only important for the regular bone structure but also for muscle strength could be demonstrated in a meta-analysis by Bischoff and colleagues who showed that a supplemental vitamin D in a dose of 700-1000 IU a day reduced the risk of falling among older individuals by 19%.^38^ This shows the importance of adequate vitamin D3 provision for the elderly population. In our own unpublished study, we showed that virtually 80% of a sample of 70 outpatients over the age of 65 had 25-hydroxyvitamin D3 concentrations below 30 ng/mL. From the age of 70 onwards, a 25-hydroxyvitamin D3 serum level of 30-70 ng/mL can be regarded as optimum. Below this range there can be a latent decline in the serum calcium concentration, which triggers secondary hyperparathyroidism with facilitation of osteoporotic changes. Furthermore, numerous studies point to the importance of adequate vitamin D3 provision for good muscle function in old age,^39^,^40^ which again is associated with the risk of frailty. It is therefore recommended, also with regard to the prophylaxis of frailty, that serum 25-hydroxyvitamin D3 concentrations to be measured in healthy people from the age of 50 onwards regularly between January and April, as 25-hydroxyvitamin D3 serum concentrations are lowest at these times. As described above, measuring the 25-hydroxyvitamin D3 serum level is the best means of detecting vitamin D3 deficiency. The LC-Tandem mass spectroscopy serves as a reference method, where the HPLC and the immunoassay should be calibrated. The immunoassay has the advantage over the chromatographic method because of the higher sample flow-rate and the lower demands on personnel and equipment. However, certain disadvantages must be taken into account, such as the non-quantitative dissolving of the analyte out of its binding protein, which presents a challenge for the assay developer. A good comparison between the various immunoassays compared to the reference method was published by Roth and colleagues.^41^ In the coming years, our knowledge about the role of the vitamin D system, especially outside bone metabolism, will continue to grow, which in the light of the wide-spread vitamin D deficiency will result in more frequent requests for analysis.

**Table 3:** Effects of 1-alpha hydroxylation in various organ systems.

Site of 1-alpha hydroxylation	Action
Kidney	Endocrine actions: bone metabolism intestinal Ca transport renal Ca transport blood pressure
Prostate, colon, breast, immune cells, beta-cells, skin	Autocrine/paracrine actions: inhibition of cell proliferation promotion of cell differentiation immune regulation

## HORMONE VALUES

The thyroid hormones play a central role in the metabolism, also with regard to the muscle function of the elderly period. Stimulated by the pituitary thyrotropin releasing hormone (TRH), there is a hypophysial secretion of the thyroid-stimulating hormone (TSH), which in turn stimulates the secretion of the thyroid hormone triiodothyronine (T3) and thyroxin (T4). These hormones react by way of a negative feedback mechanism on the secretion of TSH and TRH. Some of the clinical symptoms of a thyroid function disorder in old age are not very pronounced and often have an unspecific character.^42^ This makes clinical diagnosis difficult, especially as the clinical symptoms caused by thyroid dysfunction are often misinterpreted by the patients or their relatives as being age-related. This diagnostic dilemma justifies early examination of the TSH serum level where symptoms are unclear. If the level is pathological, the concentration of free thyroxin (fT4) and free triiodothyronine (fT3) should be measured. If these concentrations are normal and the TSH level is pathological, depending on the TSH level, this condition is referred to as latent or subclinical hyper- or hypothyroidism.^43^,^44^ If the concentrations of both thyroid hormones and also the TSH level are pathologically altered, this situation is referred to as manifest hypo- or hyperthyroidism. Adequate correction of pathological thyroid function represents an important cornerstone in avoiding and preventing the symptoms of frailty, as pathological thyroid hormone values have been demonstrated to be associated with impaired functionality in the elderly.^45^ Reference should be made in this context to articles by Weissel^46^ and Habra and Sarlis^47^ in relation to further clinical consequences and the therapeutic approach. The treatment of thyroid disease is another example to explain the “kinetic or individualistic” approach. Although there is still debate on the decision to treat or not to treat subclinical thyroid disorders, current recommendations state the necessity of considering treatment on an individual basis according to the symptomatology and to the possible benefits the older person may obtain.^48^

A further component in the hormonal system, which shows marked changes in old age, is represented by the sex hormones. The effects of oestrogen deficiency in the pathogenesis of osteoporosis with its consequences (bone fractures) are generally known and therefore are not discussed further here. Instead, the diagnosis of testosterone deficiency in men should be examined in more detail. There is a continuous decline in serum testosterone concentration in the ageing man, although it is not possible to define such a clear turning point, like the drop in oestrogen at the onset of the menopause in women. The slow, continuous loss means that the symptoms of androgen deficiency such as reduction of muscular strength, lack of drive, and increased body fat are only gradually perceived.^49^ In addition to the global reduction of testosterone, the amplitude of circadian rhythms with the highest values in the early morning, as is common in young men, declines. Testosterone is transported in the serum bound mostly to sex hormone-binding globulin (SHBG) and albumin. A number of factors affect hepatic SHBG and albumin production (hepatitis, liver cirrhosis, nephrotic syndrome), so that although the total testosterone is altered, the free, biologically active testosterone can remain in the normal range if testicular function is intact. For example, low total testosterone concentrations along with low SHBG concentrations can involve a normal concentration of free, active testosterone; so that in this case testosterone deficiency cannot be assumed. This means that the SHBG and albumin concentration should also be measured whenever testosterone is measured, particularly in patients with concomitant diseases. A calculated free testosterone (using SHBG, albumin and testosterone) can be used as a proxy for measuring free testosterone which is the active form of the hormone. It should be recognized that changes in binding characteristics of SHBG with aging make this calculation slightly inaccurate.^50^ The calculation of free testosterone is available at www.issam.ch.

The correct assessment or interpretation of the testosterone serum level is important in that it is assumed that disrupted “syncrinology” of the steroid hormones (dehydroepiandrosterone, testosterone and cortisol) in the ageing man can be regarded as a precursor of frailty^51^ and a reduced testosterone serum level results in a decline in gross muscular strength.^52^ Several age-related hormonal changes have been linked to the frailty syndrome and to its components. Among the latter, the hormonal relationship with the decrease of muscle strength received most attention. While testosterone, growth hormone and insulin-like growth factor I were most intensely studied in this context, open questions still remain with regard to clinical relevance and replacement therapy.^53^–^55^

## INFLAMMATORY CYTOKINES

A predominant role has also been attributed to inflammatory mechanisms and especially molecules from the cytokine family. Cytokines play a key role in ageing^56^ and are central factors in the pathogenesis of cachexia.^57^–^59^ Increased CRP-values, an induction product produced in the liver by interleukin-6,^60^ and proinflammatory cytokines like interleukin-6 were associated with the presence of frailty.^61^–^63^ Thus, these immune mediators possibly contribute to aggravating the comorbidities of these patients and to an increase in frailty.^64^

## OUTLOOK

By measuring the established and newer measured values presented in this article, such as cytokines, and the examination of their relationships with the functionality of the elderly, the continuing growth of knowledge about the development of frailty and the therapeutic options can be expected in the coming years. In recent years, chronic inflammatory processes associated with a reduction in muscle mass have increasingly been identified as pathogenetic components in the origin and perpetuation of frailty, and thus the hope of acquiring new knowledge which is also relevant for clinical routine practice by determining new measured values of the immune system like cytokines, does not appear to be unjustified.^65^ The article closes with this outlook on future measured values which are probably becoming important in the assessment by laboratory diagnostics and in the prevention of frailty. In conclusion, it can be stated that laboratory diagnostics, in addition to clinical observation and anthropometric data, represent an important component for the clarification of the causality of frailty and of the preventive approaches.
